# Risk factors for mortality in patients with *Stenotrophomonas maltophilia* bacteremia

**DOI:** 10.1097/MD.0000000000004375

**Published:** 2016-08-07

**Authors:** Yong Duk Jeon, Woo Yong Jeong, Moo Hyun Kim, In Young Jung, Mi Young Ahn, Hea Won Ann, Jin Young Ahn, Sang Hoon Han, Jun Yong Choi, Young Goo Song, June Myung Kim, Nam Su Ku

**Affiliations:** a Department of Internal Medicine; b AIDS Research Institute, Yonsei University College of Medicine, Seoul, Korea.

**Keywords:** bacteremia, central venous catheter, mortality, risk factor, *Stenotrophomonas maltophilia*

## Abstract

*Stenotrophomonas maltophilia* is a nosocomial pathogen associated with high morbidity and mortality, particularly in immunocompromised or critically ill patients. In this study, we investigated the risk factors for mortality in patients with *S. maltophilia* bacteremia.

Retrospectively, medical records from all patients with *S. maltophilia* bacteremia between December 2005 and 2014 at Severance Hospital, a 2000-bed tertiary care hospital in Seoul, Korea, were reviewed. Analysis was performed to identify factors associated with 28-day mortality.

In total, 142 bacteremia patients were enrolled in this study. The overall 28-day mortality rate was 36.6%. Based on the univariate analysis, hematologic malignancy (*P* = 0.015), Sepsis-related Organ Failure Assessment (SOFA) score (*P* < 0.001) and the removal of a central venous catheter (CVC) (*P* = 0.040) were significantly related to mortality. In the intensive care unit patients, the Acute Physiology and Chronic Health Evaluation II score (*P* = 0.001) also had significance. Based on the multivariate analysis, the SOFA score (odds ratio [OR] = 1.323; 95% confidence interval [CI]: 1.159, 1.509; *P* < 0.001) and removal of the CVC (OR = 0.330; 95% CI: 0.109, 0.996; *P* = 0.049) were independent factors associated with mortality.

Our results suggest that removing a CVC may considerably reduce mortality in patients with *S. maltophilia* bacteremia.

## 1 Introduction

*Stenotrophomonas maltophilia* is a glucose non-fermentative, Gram-negative bacillus that has the ability to colonize epithelial cells of the respiratory tract and surfaces of medical devices.^[1]^ It has inherent resistance to several antibiotics such as carbapenem, and the increasing use of antibiotics has allowed this bacterium to become a predominant nosocomial pathogen.^[2]^

Predisposing factors of *S. maltophilia* infection are well known and include CVCs, urinary catheters, mechanical ventilation, recent surgery, malignancies, admission to an intensive care unit (ICU), immunosuppressive drugs, neutropenia, and prior antibiotic use.^[3,4]^ Pneumonia and bacteremia are the most common clinical manifestations of *S. maltophilia* infection. Less frequently, it can cause urinary tract infections, cholangitis, peritonitis, wound infections, eye infections, arthritis, meningitis, and endocarditis.^[1]^

*S. maltophilia* is associated with high morbidity and mortality, ranging from 21 to 69%, and is particularly observed in immunocompromised or critically ill patients.^[5,6]^ Treatment of *S. maltophilia* infection can be difficult because of its inherent resistance to a variety of antibiotics.^[7,8]^ For *S. maltophilia*, trimethoprim–sulfamethoxazole (TMP–SMX) is the drug of choice, and fluoroquinolone is the proposed alternative.

Several studies have reported the risk factors for mortality associated with *S. maltophilia* infection.^[2,4,9–18]^ However, these studies have not shown consistent results, with some being contradictory to others. Furthermore, it is difficult to distinguish between *S. maltophilia* colonization and infection, which can affect the results of studies focused on identifying the risk factors for mortality.^[3]^ Therefore, in our study, we utilized patients with *S. maltophilia* bacteremia to investigate the risk factors for mortality.

## 2 Methods

### 2.1 Study population and design

A retrospective cohort study was conducted to evaluate the risk factors for morality in *S. maltophilia* bacteremia at Severance Hospital, a tertiary care hospital in Seoul, Korea. All the patients were >18 years of age and tested positive for *S. maltophilia* in 1 or more blood cultures between December 2005 and 2014. For patients that had more than 1 episode of *S. maltophilia* bacteremia, only the first episode was accepted. Clinical and laboratory data were collected from electronic medical records, including 28-day mortality. The Sepsis-related Organ Failure Assessment (SOFA) score was calculated in all patients, and the Acute Physiology and Chronic Health Evaluation (APACHE) II score was calculated in ICU patients. This study was approved by the Institutional Review Board and local Ethics Committee of Severance hospital.

### 2.2 Definitions

*S. maltophilia* bacteremia was defined as a patient having 1 or more positive blood culture, combined with clinical symptoms of systemic inflammatory response syndromes.^[11]^ Polymicrobial bacteremia was defined as 2 or more bacterial species identified in multiple blood culture samples collected within 24 hours.^[15]^ Nosocomial bacteremia was defined as occurring ≥48 hours after admission. Healthcare-associated bacteremia was defined as bacteremia that occurs in a patient who has stayed in a nursing home, has been admitted to a hospital within the previous month, received hemodialysis, or has been treated as an outpatient with intravenous antibiotics or chemotherapy within the previous 2 weeks. Community-acquired bacteremia was defined as bacteremia that occurred within 48 hours of admission and in patients who did not meet the criteria for healthcare-associated bacteremia.^[10]^ The source of bacteremia was determined if there was an active site of infection, and *S. maltophilia* was identified from that site immediately before—or the same day of—bacteremia onset.^[15]^ When blood cultures from the periphery and CVC both tested positive for *S. maltophilia* in the absence of other active sites of infection, we defined this as a catheter-related infection. Prior antibiotic use was defined as any antibiotic treatment for more than 24 hours within 1 month before the episode of bacteremia.^[19]^ Immunosuppressive therapy was defined as a daily dose of ≥10 mg prednisolone-equivalent steroid, monoclonal antibodies, antimetabolite drugs, or T-cell inhibitors within 30 days before bacteremia onset.^[20]^ Neutropenia was defined as an absolute neutrophil count of <500/mm^3^ at the onset of bacteremia.^[6]^ Empirical antibacterial therapy was defined as treatment that was initiated no later than 24 hours after blood cultures were drawn. Definitive antibacterial therapy was defined as treatment that was continued or commenced after blood culture results were reported and that was started no later than 120 hours after blood cultures were drawn.^[21]^ Antibacterial therapy was regarded as appropriate if the targeted regimen included at least 1 antibiotic agent to which *S. maltophilia* was susceptible in vitro.^[20]^ The removal of the CVC was defined when this was performed no later than 5 days after blood cultures were drawn.

### 2.3 Clinical techniques

Either conventional bacterial isolation techniques or the ATB 32 GN system (bioMérieux, Marcy l’Etoile, France) was used to evaluate clinical isolates. Antimicrobial susceptibility tests were performed using the disk-diffusion method or a VITEK-2 N131 card (bioMerieux, Hazelwood, MO). The results were interpreted on the basis of the Clinical and Laboratory Standards Institute guidelines.

### 2.4 Statistical analysis

Data were analyzed using SPSS for Windows (ver. 20.0, SPSS Inc., Chicago, IL). Analysis was performed to assess the factors associated with 28-day mortality. Student *t* test was used for continuous variables and the Chi-square test or Fisher exact test was used for categorical variables. A two-sided *P* value <0.05 was considered to be statistically significant. Multiple logistic regressions were performed to determine independent risk factors for 28-day mortality, and the Kaplan–Meier method was utilized for survival analysis.

## 3 Results

### 3.1 Demographic characteristics

In total, 142 bacteremia patients were enrolled in this study. Patient demographics are presented in Table 1. The median age was 61 years, and 60 patients (42.3%) were >65 years. There were 88 male (62.0%) and 54 female (38.0%) patients. The most frequent underlying conditions were solid tumor (55.6%), hematologic malignancy (23.2%), and diabetes mellitus (16.2%). Polymicrobial bacteremia occurred in 38 patients (26.8%). Common pathogens that were found concurrently with *S. maltophilia* included enterococci in 12 patients, coagulase negative staphylococci in 6 patients, *Acinetobacter* spp. in 6 patients, *Serratia* spp. in 4 patients, *Candida* spp. in 3 patients, and *Pseudomonas* spp. in 3 patients. In 142 episodes of bacteremia, 125 episodes (88.7%) were nosocomial, 14 (14%) were healthcare-associated, and 2 (1.4%) were community-acquired. Common sources of bacteremia included the respiratory tract in 48 patients (33.8%), CVC in 35 patients (24.6%), and biliary tract in 32 patients (22.5%). Forty-four patients (31.0%) were in the ICU when bacteremia occurred. There were 14 patients (9.9%) who received appropriate empirical antibacterial therapy and 55 (38.7%) that received appropriate definitive antibacterial therapy. There were 21 patients (14.8%) treated with TMP–SMX and 40 (28.2%) treated with levofloxacin. A CVC was used in 95 patients (66.9%) and was removed from 27/95 patients (28.4%). The mean time from drawing the blood cultures to removal was 2.3 ± 1.1 days. The 28-day mortality rate was 36.6% (52/142).

**Table 1:**
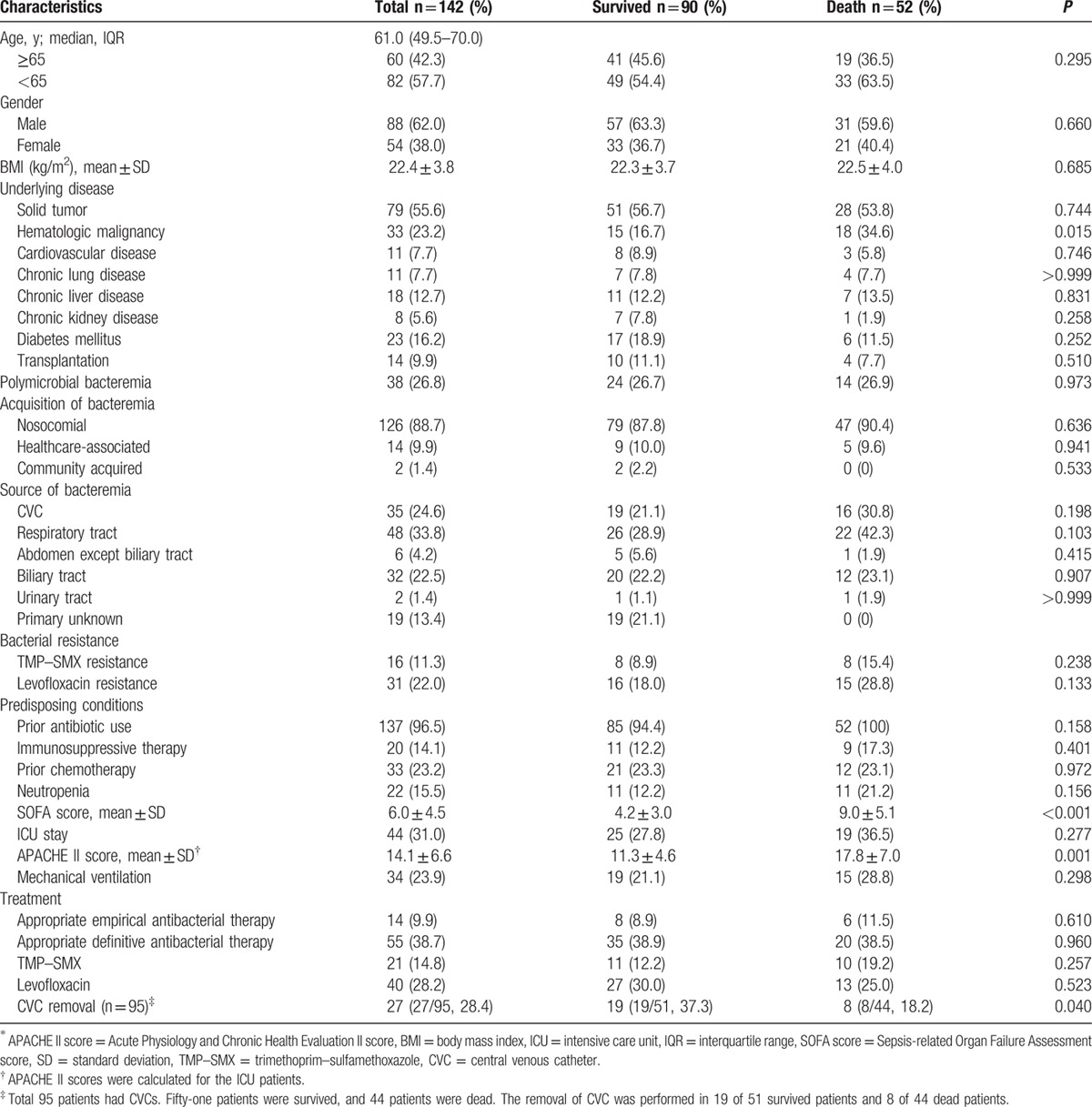
Overall characteristics and univariate analysis of 28-day mortality in patients with *S. maltophilia* bacteremia.

### 3.2 Antimicrobial susceptibilities

From the patients’ blood cultures, 16 isolated *S. maltophilia* strains (11.3%) were resistant to TMP–SMX and 31 strains (22.0%) were resistant to levofloxacin.

### 3.3 Risk factors for mortality in patients with *S. maltophilia* bacteremia

**Figure 1:**
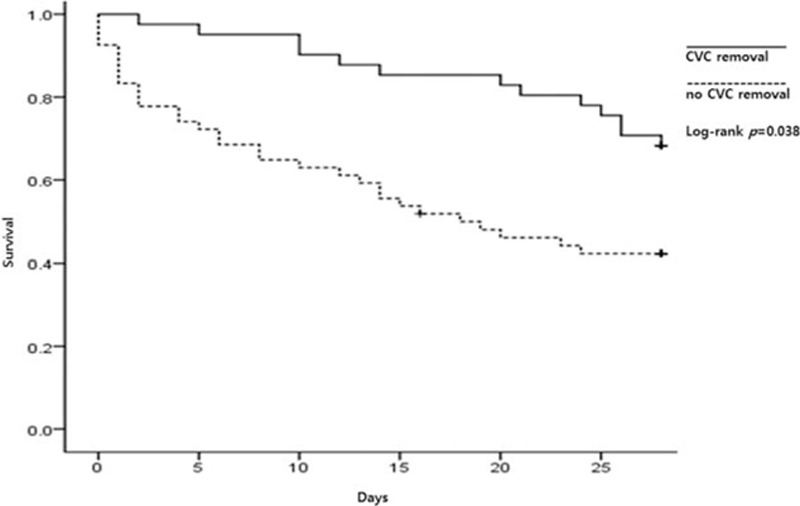
Kaplan–Meier curve comparing survival between patients with and without the removal of the central venous catheter. ^∗^CVC = central venous catheter.

A univariate analysis was performed to identify risk factors associated with 28-day mortality (Table 1). Hematologic malignancy (*P* = 0.015), SOFA score (*P* < 0.001), and removal of the CVC (*P* = 0.040) were significantly related to mortality. In the ICU patients, the APACHE II score (*P* = 0.001) also had significance. Based on the multivariate analysis, the SOFA score (odds ratio [OR] = 1.323; 95% confidence interval [CI]: 1.159, 1.509; *P* < 0.001) and removal of the CVC (OR = 0.330; 95% CI: 0.109, 0.996; *P* = 0.049) were independent factors associated with mortality (Table 2). Hematologic malignancy was not a significant independent factor when combined in the logistic regression model. In addition, a Kaplan–Meier curve was drawn to estimate the impact of CVC removal on survival (Fig. 1). Patients who had the CVC removed had significantly higher survival rates compared to those who did not have the catheter removed (log-rank *P* = 0.038).

**Table 2:**
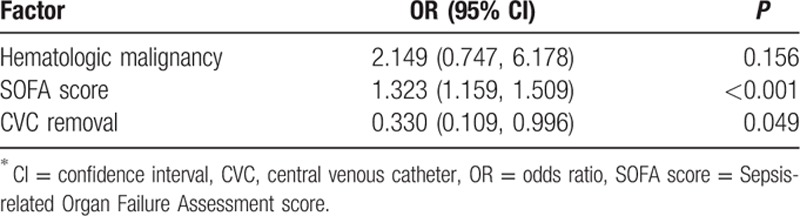
Multivariate analysis of factors associated with 28-day mortality in patients with *S. maltophilia* bacteremia.

## 4 Discussion

Our results show that *S. maltophilia* bacteremia patients with high SOFA scores had higher rates of mortality, and that removal of the CVC was a protective method to reduce mortality. Several studies have focused on the risk factors for mortality of *S. maltophilia* bacteremia but have utilized small sample sizes.^[5,6,10,11,13,15–17,19,20]^ In this study, we enrolled 142 patients; to date, this is the largest study investigating risk factors for mortality in patients with *S. maltophilia* bacteremia.

In our study, we identified SOFA score as an independent risk factor associated with mortality, based on the results of our multivariate analysis. Several studies have reported similar results, as well as reporting that patient medical conditions were more important factors associated with mortality than the appropriateness of antibiotics.^[10,13,22,23]^ In addition, our results show that the appropriateness of antibiotics, as well as empirical and definitive antibacterial therapy, was not associated with mortality. However, other studies have reported that the appropriate antibacterial therapy is significantly associated with lower mortality.^[5,24,25]^ Further investigation to evaluate the impact of appropriate antibacterial therapy should be performed.

We observed that the removal of the CVC was significantly associated with lower mortality, based on the results of our multivariate analysis. Previous studies reported that many patients with *S. maltophilia* bacteremia had CVCs because *S. maltophilia* bacteremia occurs mainly in immunocompromised or critically ill patients.^[6,10]^ In addition, it is known that CVCs may be a risk factor for *S. maltophilia* infections.^[1]^ In our study, 95 patients (66.9%) had a CVC and 27/95 patients (28.4%) had it removed. Some reports have suggested that the removal of the CVC in patients with *S. maltophilia* bacteremia is beneficial.^[15,19,24,26]^ More specifically, 3 studies have reported the implications of removing the CVC in catheter-related infections.^[15,19,26]^ However, our results suggest that removal was related with lower mortality irrespective of the bacteremia source in patients who had a CVC. Other studies have reported similar results and suggested that this may imply colonization rather than true source of bacteremia in cases in which *S. maltophilia* was isolated from other sources like the respiratory tract. These results suggest that in other cases, the CVC may be the true source of infection.^[24]^ Furthermore, we believe that the CVC may be an additional secondary source, although it was not the primary source. Our study could strengthen the evidence supporting the removal of the CVC to reduce mortality in all bacteremia patients regardless of the presence of a catheter-related infection.

In this study, common underlying conditions were solid tumor (55.6%) and hematologic malignancy (23.2%). These results are consistent with other studies, which indicate that these conditions are associated with immunocompromised status after chemotherapy, use of medical devices, exposure to antibiotics, and long hospital stays in cancer patients.^[1]^ Based on the univariate analysis, hematologic malignancy (*P* = 0.015) was significantly related to mortality, but no significance (*P* = 0.156) was observed in multivariate analysis. In 14 cases (9.9%), the patient was a transplant recipient. As transplantation is increasing globally, this could mean an increase in patients prone to various infections including *S. maltophilia*.^[27]^

Common sources of bacteremia were the respiratory tract (33.8%), CVC (24.6%), and biliary tract (22.5%). The respiratory tract and CVC s are well-known sources of *S. maltophilia* infections.^[1]^ The biliary tract was the source of bacteremia in a large number of patients, and this may be due to percutaneous transhepatic biliary drainage (PTBD) catheters. All patients whose source of bacteremia was the biliary tract had PTBD catheters, suggesting that this medical device may be a risk factor for *S. maltophilia* infections.

Our study had several limitations. First, we included patients from a single center, which may make it difficult to apply our results to other hospitals. Second, the design of our study was retrospective. This type of study may have selection and information biases. Third, the true source of bacteremia was not easy to identify because it was not easy to distinguish between colonization and infection when *S. maltophilia* was identified from the sites other than blood. This would be related to the possibility of identifying the incorrect source of infection. Lastly, we used all-cause mortality as the primary end point, and this indicates that the effect of underlying disease or other medical condition was not excluded.

In conclusion, our results suggest that removal of the CVC should be considered to reduce mortality in patients with *S. maltophilia* bacteremia. In addition, we believe a well-designed study to evaluate the impact of appropriate antibacterial therapy is required.
